# Mechanisms of Transcranial Doppler Ultrasound phenotypes in paediatric cerebral malaria remain elusive

**DOI:** 10.1186/s12936-022-04163-0

**Published:** 2022-06-21

**Authors:** Nicole F. O’Brien, Yudy Fonseca, Hunter C. Johnson, Douglas Postels, Gretchen L. Birbeck, Yamikani Chimalizeni, Karl B. Seydel, Montfort Bernard Gushu, Tusekile Phiri, Sylvester June, Karen Chetcuti, Lorenna Vidal, Manu S. Goyal, Terrie E. Taylor

**Affiliations:** 1 Department of Pediatrics, Division of Critical Care Medicine, Nationwide Children’s Hospital, The Ohio State University, 700 Children’s Drive, Columbus, OH 43502 USA; 2 Department of Neurology, George Washington University/Children’s National Medical Center, Washington, DC USA; 3 Department of Neurology, University of Rochester, Rochester, NY USA; 4 University Teaching Hospitals Children’s Hospital, Lusaka, Zambia; 5 Department of Pediatrics and Child Health, Kamuzu University of Health Sciences, Chichiri, Blantyre 3, Malawi; 6 Dept of Osteopathic Medical Specialties, College of Osteopathic Medicine, Michigan State University, East Lansing, MI 48824 USA; 7 Queen Elizabeth Central Hospital, The Blantyre Malaria Project, Private Bag 360, Chichiri, Blantyre 3, Malawi; 8 Department of Radiology, Division of Neuroradiology, Children’s Hospital of Philadelphia, University of Pennsylvania, Philadelphia, PA 19104 USA; 9 Washington University School of Medicine, St. Louis, MO USA

**Keywords:** Paediatric, Cerebral malaria, Transcranial Doppler Ultrasound, Cerebral blood flow

## Abstract

**Background:**

Cerebral malaria (CM) results in significant paediatric death and neurodisability in sub-Saharan Africa. Several different alterations to typical Transcranial Doppler Ultrasound (TCD) flow velocities and waveforms in CM have been described, but mechanistic contributors to these abnormalities are unknown. If identified, targeted, TCD-guided adjunctive therapy in CM may improve outcomes.

**Methods:**

This was a prospective, observational study of children 6 months to 12 years with CM in Blantyre, Malawi recruited between January 2018 and June 2021. Medical history, physical examination, laboratory analysis, electroencephalogram, and magnetic resonance imaging were undertaken on presentation. Admission TCD results determined phenotypic grouping following a priori definitions*.* Evaluation of the relationship between haemodynamic, metabolic, or intracranial perturbations that lead to these observed phenotypes in other diseases was undertaken. Neurological outcomes at hospital discharge were evaluated using the Paediatric Cerebral Performance Categorization (PCPC) score.

**Results:**

One hundred seventy-four patients were enrolled. Seven (4%) had a normal TCD examination, 57 (33%) met criteria for hyperaemia, 50 (29%) for low flow, 14 (8%) for microvascular obstruction, 11 (6%) for vasospasm, and 35 (20%) for isolated posterior circulation high flow. A lower cardiac index (CI) and higher systemic vascular resistive index (SVRI) were present in those with low flow than other groups (p < 0.003), though these values are normal for age (CI 4.4 [3.7,5] l/min/m2, SVRI 1552 [1197,1961] dscm-5m2). Other parameters were largely not significantly different between phenotypes. Overall, 118 children (68%) had a good neurological outcome. Twenty-three (13%) died, and 33 (19%) had neurological deficits. Outcomes were best for participants with hyperaemia and isolated posterior high flow (PCPC 1–2 in 77 and 89% respectively). Participants with low flow had the least likelihood of a good outcome (PCPC 1–2 in 42%) (p < 0.001). Cerebral autoregulation was significantly better in children with good outcome (transient hyperemic response ratio (THRR) 1.12 [1.04,1.2]) compared to a poor outcome (THRR 1.05 [0.98,1.02], p = 0.05).

**Conclusions:**

Common pathophysiological mechanisms leading to TCD phenotypes in non-malarial illness are not causative in children with CM. Alternative mechanistic contributors, including mechanical factors of the cerebrovasculature and biologically active regulators of vascular tone should be explored.

## Background

There were an estimated 241 million malaria cases and 627,000 deaths worldwide in 2020 [1].

The burden is heaviest in sub-Saharan Africa, where 94% of the deaths occur, primarily in children younger than 5 years of age. Cerebral malaria (CM) is a severe manifestation of the disease with case fatality rates of 15–40%, even with effective treatment [2–4]. Deficits in gross motor or sensory function, cognition, behavior, and/or subsequent epilepsy occur in more than 50% of survivors [5, 6]. As such, CM is a leading cause of death and disability in African children [3]. While magnetic resonance imaging has provided improved understanding of the anatomic abnormalities that occur in paediatric CM, pathohysiological contributors to these neuroradiologic findings remain less clear [2, 7, 8]. In order to develop efficacious adjunctive therapeutic approaches that improve outcomes in CM, mechanisms of neurological injury must determined.

Transcranial Doppler Ultrasound (TCD) is a portable, non-invasive method to assess the cerebral blood flow velocities (CBFVs) and haemodynamics in the major cerebral vessels [9–14]. TCD derived CBFVs and morphologic waveforms are determined by the mean arterial pressure, the tone and patency of the systemic and cerebral vessels, and the intracranial and central venous pressures [15–21]. Alterations to any of these factors results in distinct changes to measured TCD parameters and waveform morphology (Table 1). Thus, TCD is used as a point of care tool to determine specific mechanisms of focal or global cerebral dysfunction in several clinical scenarios [22–29]. Therefore, TCD may also be of aid in determining mechanisms of neurological injury in CM.

**Table 1:**
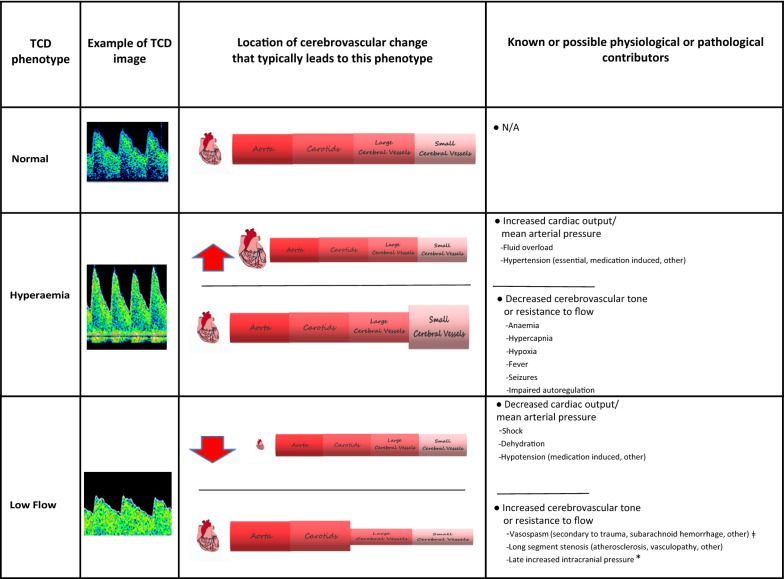

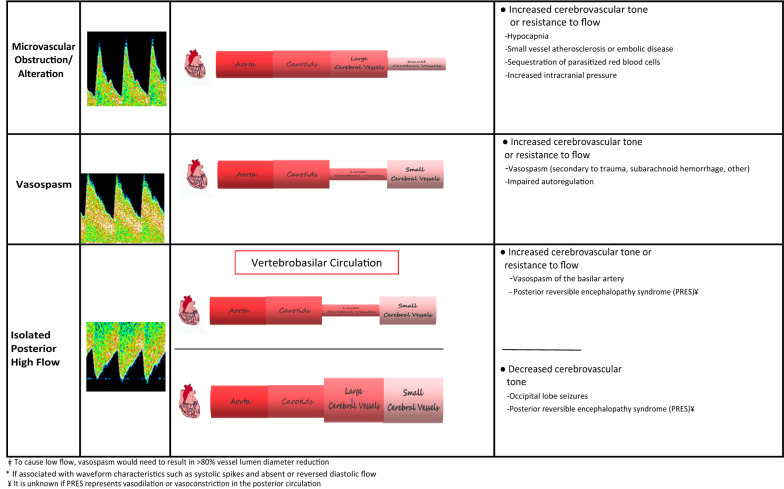
Physiological or pathological factors that contribute to Transcranial Doppler Ultrasound (TCD) flow velocity or waveform alterations

TCD findings are dependent on cerebral perfusion pressure (CPP) and inversely proportional to the cerebrovascular resistance (CVR) [15–21]

ǂTo cause low flow, vasospasm would need to result in > 80% vessel lumen diameter reduction

^*^If associated with waveform characteristics such as systolic spikes and absent or reversed diastolic flow

¥It is unknown if PRES represents vasodilation or vasoconstriction in the posterior circulation

Five different patterns of abnormal TCD flow velocities and waveforms have been observed in children with CM in the Democratic Republic of the Congo [30]. Serial assessments showed that the admission phenotype was generally sustained until the patient recovered or died. TCD phenotypes were also associated with distinct risks of neurological morbidity or mortality. Evaluation of the association between mechanisms that commonly lead to these observed phenotypes in non-CM illness was not done.

This prospective, observational study was performed to evaluate the presence and frequency of each TCD phenotype in Malawian children with CM. Additionally, the associations of common pathophysiological mechanisms known to contribute to each phenotype in other disease states were assessed. If mechanisms are determined, TCD may be used as a bedside tool to direct, in real-time, individualized mechanism-specific adjunctive therapy in CM.

## Methods

Malawi is a low-income country in sub-Saharan Africa with a population of approximately 18 million people, including over three million children under 5 years of age. Queen Elizabeth Central Hospital (QECH) is a 1250-bed public tertiary hospital in Blantyre, Malawi, with a catchment area of approximately six million people in the southern part of the country. This prospective, observational study was performed from January 2018 to June 2021 at QECH in conjunction with the “Treating Brain Swelling in Paediatric Cerebral Malaria” clinical trial (5U01AI126610-02, ClinicalTrials.gov NCT03300648). The study was approved by the ethics committee at Michigan State University and at the University of Malawi College of Medicine Research Ethics Committee (COMREC). All subjects’ guardians provided verbal and written informed consent.

Children 6 months to 12 years of age who met the World Health Organization case definition of cerebral malaria (*Plasmodium falciparum* parasitaemia, Blantyre Coma Score (BCS) ≤ 2, and no other discernable cause of encephalopathy) were approached for enrollment [1]. Direct and indirect ocular fundoscopy was performed at admission, and patients were subgrouped as retinopathy positive or negative based on the presence or absence of characteristic retinal findings previously reported in CM [31]. Children with sickle cell disease (known or suspected) were excluded, given the high frequency of abnormal TCD examinations in this population. Likewise, given the unknown impact of severe malnutrition (mid-upper arm circumference < 11 cm) or advanced HIV disease (known HIV positive status with severe wasting) on TCD examinations, these children also were excluded.

Demographic data, vital signs, and physical examination findings were collected. Finger-prick samples were analysed to determine parasite species and density, packed-cell volume, and blood glucose and lactate concentrations (Aviva Accu-Check, Zurich, Switzerland and Arkray Lactate Pro 2, Kyoto, Japan). Finger-prick samples were also obtained for blood gas analysis (Abbot iSTAT, Chicago, Illinois, USA). Venous blood was drawn to obtain a complete blood count and electrolyte analysis (Coulter Counter; Beckman Coulter, Brea, California, USA). Parasite counts, packed-cell volume, glucose, and lactate concentrations were evaluated every six hours until the BCS was 5 or for 72 h, whichever came later. An admission lumbar puncture was performed, opening pressure measured, and the cerebrospinal fluid was analysed [32, 33]. All patients underwent an admission electroencephalogram (EEG)(Ceegraph digital machine, BioLogic, Natus Medical Incorporated, Pleasanton, California, USA) with a modified 10–20 system to evaluate for non-convulsive status epilepticus. EEGs were clinically reviewed by a neurologist with fellowship training in EEG to evaluate for seizures/subclinical status epilepticus. When imaging capabilities were available (2018–2019 and March-June 2021), participants underwent a brain magnetic resonance imaging (MRI)(0.35-T Signa Ovation Excite, General Electric, Boston, Massachusetts or 0.064-T Hyperfine Swoop® Guilford, Connecticut, USA) to evaluate the brain volume [2]. MRIs were systematically reviewed by radiologists experienced in radiographic findings of children with CM. All patients underwent daily, non-invasive, evaluation of their systemic haemodynamics including cardiac output, cardiac index (CI), stroke volume, stroke volume index (SVI), and systemic vascular resistance using a handheld portable ultrasound device (Butterfly IQ, Guilford, CT, USA). CI was calculated as = Heart rate x SVI and SVI as = End diastolic volume – End systolic volume. Optic nerve sheath diameter was also measured daily (Butterfly IQ, Guilford, CT, USA).

All patients received intravenous artesunate according to national guidelines. Patients received 20 mL/kg of whole blood if admission packed cell volume was < 15% or > 15% but with signs of intolerance (defined as respiratory distress or haemodynamic compromise with capillary refill time > 2 s, weak pulse, and/or cool extremities). Intravenous dextrose (1 mL/kg of Dextrose 50%) was given when documented hypoglycaemia occurred (< 3 mmol/L). Clinical or sub-clinical seizure activity identified on EEG was treated with 0.2 mg/kg of diazepam followed by phenobarbital 20 mg/kg.

### TCD examinations

TCD was performed using a commercially available unit (NovaSignal, Los Angeles, California, USA). One limitation of TCD is that it is operator dependent with diagnostic accuracy depending on the skill and experience of the examiner. All study personnel who performed TCD examinations for this study participated in 10 h of online didactic training, completed > 50 proctored TCD examinations, and demonstrated a coefficient of variation < 10% for each study measurement compared to the trainer (author NO) before being considered proficient for independent TCD scanning.

The initial TCD examination occurred within 4 h of admission. TCD was performed after initial blood and dextrose infusions, if prescribed, were complete. Middle cerebral arteries (MCAs), extracranial internal carotid arteries (Ex-ICA), and basilar arteries were insonated at 2-mm intervals using previously described methods [9–14]. Systolic (Vs), diastolic (Vd), and mean flow (Vm) velocities were recorded at each interval. Pulsatility index (PI = (Vs-Vd/Vm)), a marker of downstream cerebrovascular resistance (CVR), was automatically calculated by the TCD unit at each depth in each vessel. To differentiate causes of high CBFV values, the Lindegaard ratio (LR = MCA Vm/Ex-ICA Vm) was calculated [11]. A LR < 3 was considered to represent hyperaemia whereas a LR > 3 was considered to represent vascular narrowing. Autoregulation is the capacity of the cerebrovasculature to maintain constant cerebral blood flow over a wide range of mean arterial blood pressures. Autoregulation can be impaired or lost in several clinical scenarios. Therefore, the transient hyperemic response ratio (THRR) was used to interrogate cerebral autoregulation in study subjects [12]. THRR < 1.1 represented impaired autoregulation and ≥ 1.1 represented intact autoregulation. Based on the admission TCD findings, subjects were classified into the following phenotypes: normal, hyperaemia, low flow, microvascular obstruction, vasospasm, isolated posterior circulation high flow, or terminal intracranial hypertension (Table 2). Participants underwent daily TCD examinations through discharge, death, or hospital day 8, whichever came later.

**Table 2:** Definitions used to categorize participants into Transcranial Doppler Ultrasound phenotypes

Normal Flow
(1) Systolic, diastolic, and mean flow velocity in the middle cerebral artery ± 2 standard deviations (SD) from the age normal value
Hyperaemia
(1) Systolic, diastolic, and mean flow velocity in the middle cerebral artery ≥ 2 SD above the age normal value AND
(2) Lindegaard ratio (LR)^a^ < 3
Low flow
(1) Systolic, diastolic, and mean flow velocity in the middle cerebral artery ≤ 2 SD below the age normal value AND
(2) Pulsatility index (PI)^b^ < 1.2
Microvascular obstruction/alteration
(1) Systolic flow velocity in middle cerebral artery within 2 SD of the age normal value AND
(2) Diastolic flow velocity in middle cerebral artery ≤ 2 SD below the age normal value AND
(3) PI ≥ 1.2
Vasospasm
(1) Mean flow velocity in the middle cerebral artery ≥ 2 SD above the age normal value AND
(2) LR ≥ 3
Isolated posterior high flow
(1) Mean flow velocity in the basilar artery ≥ 2 SD above the age normal value AND
(2) Mean flow velocity in both middle cerebral arteries within 2 SD of the age normal value
Terminal intracranial hypertension
(1) Systolic flow velocity in the middle cerebral artery ≤ 2 SD below the age normal value WITH associated systolic spikes on waveform analysis AND
(2) Absence of or reversal of diastolic flow

^a^Lindegaard Ratio (LR) = (Mean flow velocity in the middle cerebral artery/ mean flow velocity in the extra-cranial carotid artery)

^b^Pulsatility Index (PI) = (Systolic flow velocity-Diastolic flow velocity/Mean flow velocity)

### Outcomes

The Paediatric Cerebral Performance Category (PCPC) scoring system is a tool that was developed to measure and quantify morbidity after paediatric critical illness [34, 35]. Scores range from 1 to 6, with 1 being a normal functional level and 6 being death. Other values represent progressive impairment: 2 = mild disability (alert and able to interact at an age appropriate level but with mild cognitive, behavioral, or neurological deficits), 3 = moderate disability (alert and able to carry out age appropriate activities of daily life but with obvious cognitive or neurological deficits that limit function), 4 = severe disability (conscious but dependent on others for all daily functions), and 5 = vegetative state (any degree of coma or an inability to interact with the environment). PCPC was scored at the time of hospital discharge. Children with a PCPC of 1 or 2 were considered to have a good outcome while those with a PCPC of 3 to 6 were considered to have a poor outcome.

### Statistical analyses

Variables were summarized using medians with interquartile ranges and frequencies with percentages. Differences by phenotype were explored using Kruskal–Wallis tests, with Dwass, Steel, Critchlow-Fligner corrections for multiple comparisons for continuous and ordinal variables, and chi-square or Fisher’s exact tests for categorical variables. All analyses were conducted using R for Statistical Computing and SAS 9.4.

## Results

**Fig. 1:**
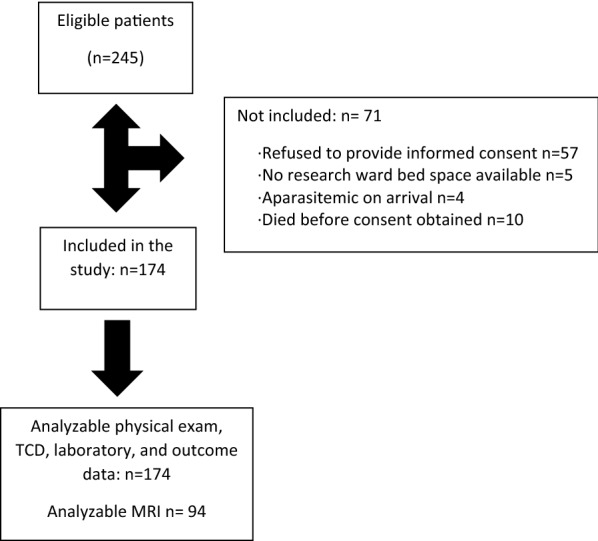
Flow diagram of patient screening and enrollment

A total of 245 potential participants were screened and 174 were enrolled (Fig. 1). Demographics, admission physical examination findings, and admission laboratory results are summarized in Table 3.

**Table 3:** Demographics, laboratory investigations, imaging, and outcomes for the cohort (n = 174)

Variable	Value
Demographics	
Age (months), mean (SD)	58 (± 32)
Male, n (%)	97 (56)
Vital signs	
Temperature (°C), median [IQR]	38.3 [37.4,39.1]
Heart rate (beats/min), median [IQR]	140 [126,157]
RR (breaths/min), median [IQR]	34 [29, 42]
Oxygen saturation (%), median [IQR]	97 [96,98]
MBP (mmHg), median [IQR]	77 [71,86]
Hemodynamic findings	
Pulse pressure (mmHg), median [IQR]	40 [35, 45]
Stroke volume index (ml/m^2^), median [IQR]	38 [32, 44]
Cardiac index (l/min/m^2^), median [IQR]	5 [4.3,5.8]
Systemic vascular resistive index (d.s.cm^−5^m^2^), median [IQR]	1314 [1152,1630]
Laboratory investigations	
Packed Cell Volume (%), median [IQR]	26 [22, 30]
Glucose (mmol/L), median [IQR]	5.65 [4.5, 6.5]
Lactate (mmol/L), median [IQR]	4.2 [2.3,6.9]
Parasites/microliter blood, median [IQR]	255,000 [9300,665000]
PfHRP2 (ng/mL), median [IQR]	571 [219,1332]
pH, median [IQR]	7.41 [7.35,7.46]
CO_2_ (mmHg), median [IQR]	28 [24, 33]
Base excess, median [IQR]	− 5 [− 9,− 2]
Sodium (mEq/L), median [IQR]	138 [135,143]
Bicarbonate (mmol/L), median [IQR]	17 [13, 20]
Clinical Features	
Retinopathy positive, n (%)	115 (66%)
Blantyre coma score, n (%)	
0	26 (15%)
1	64 (37%)
2	84 (48%)
Seizures on EEG, n (%)	16 (9%)
Papilloedema present, n (%)	10 (7%)
Opening pressure (cm H20), median [IQR]	17 [12, 22]
ONSD (mm), median [IQR]	4.8 [4.4,5.1]
Time to coma resolution (hrs), median [IQR]	38 [22,70]
Magnetic resonance imaging findings (n = 94)ǂ	
Brain volume score, n (%)	
3	7 (7%)
4	8 (8%)
5	33 (35%)
6	24 (26%)
7	18 (19%)
8	4 (5%)
Outcome	
Good, n (%)	
PCPC 1–2 (normal, mild disability)	118 (68%)
Poor, n (%)	
PCPC 3–5 (moderate to severe disability)	33 (19%)
PCPC 6 (died)	23 (13%)

*n* number, *SD* standard deviations, *IQR* interquartile range, *hrs* hours, *RR* respiratory rate, *MBP* mean blood pressure, *mmHg* millimeters mercury, *SVRI* systemic vascular resistive index, *PfHRP2* Plasmodium falciparum histidine rich protein 2, *CO*_2_ carbon dioxide, *NIRS* near-infrared spectroscopy, *SO*_2_ cerebral oxygen saturation, *EEG* electroencephalogram, *OP* opening pressure, *ONSD *optic nerve sheath diameter, *MRI* magnetic resonance imaging, *TCD* transcranial doppler ultrasound, *CSF* cerebrospinal fluid

ǂMRI not available for all participants

### Transcranial Doppler Ultrasound examinations

**Fig. 2:**
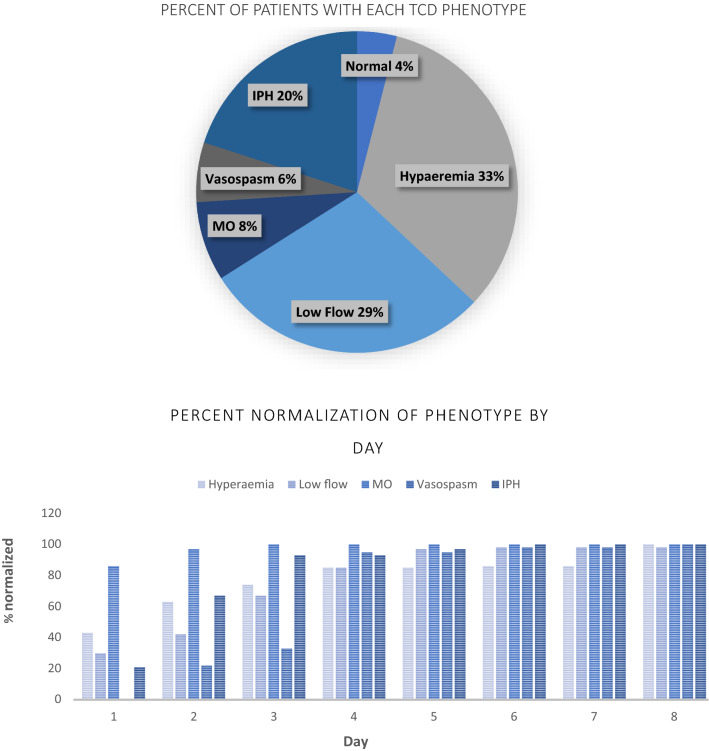
Frequency and time to normalization of each Transcranial Doppler Ultrasound phenotype. Only survivors are included in the time to normalization graph. *TCD* transcranial doppler ultrasound, *MO* microvascular obstruction, *IPH* isolated posterior circulation high flow

**Fig. 3:**
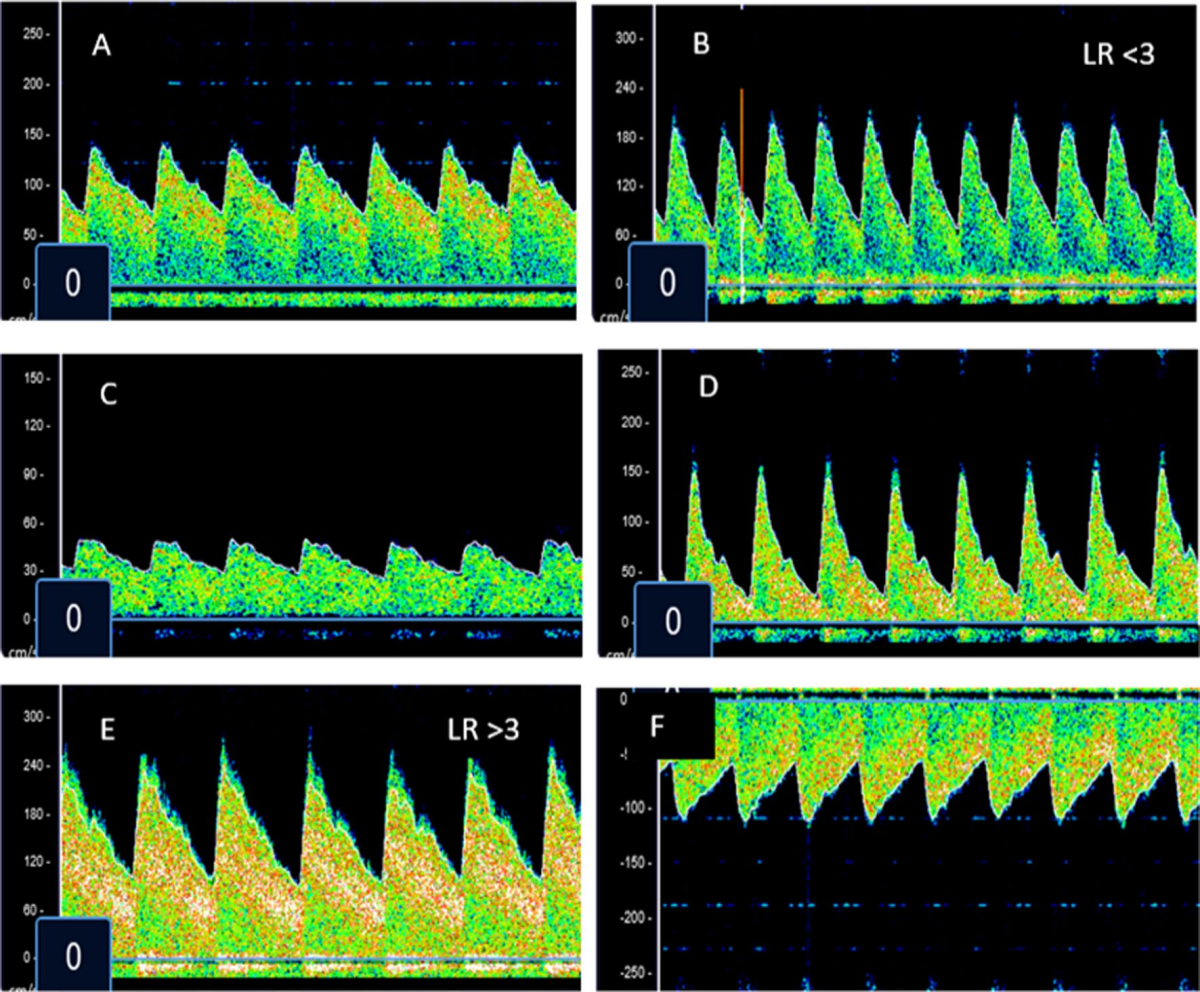
Representative images of Transcranial Doppler Ultrasound phenotypes in children with cerebral malaria. **A** Normal middle cerebral artery (MCA) TCD flow velocities and waveform for a 3-year-old child. **B** TCD with increased systolic flow velocity, increased diastolic flow velocity, Lindegaard ratio (LR) < 3. These findings represent a child categorized as having hyperaemia. **C** TCD with decreased systolic flow velocity, decreased diastolic flow velocity, decreased mean flow velocity. These findings represent a child categorized as having low flow. **D** TCD with normal systolic flow velocity, reduced diastolic flow velocity, increased pulsatility index. These findings represent a child categorized as having microvascular obstruction. **E** TCD with increased systolic flow velocity, increased diastolic flow velocity, LR > 3. These findings represent a child categorized as having cerebral vasospasm. **F** TCD with increased systolic flow velocity, increased diastolic flow velocity, increased mean flow velocity in the basilar artery. At the same time, all measurements in the MCAs were normal. These findings represent a child categorized as having isolated posterior circulation high flow

On admission, seven children (4%) had a normal TCD examination. Fifty-seven children (33%) met criteria for hyperaemia, 50 (29%) for low flow, 14 (8%) for microvascular obstruction, 11 (6%) for vasospasm, and 35 (20%) for isolated high flow in the posterior circulation (Fig. 2). No participant met criteria for terminal intracranial hypertension on the admission TCD. Eleven participants (7%) transitioned from one phenotype to another on subsequent evaluation: 5 with isolated posterior high flow (IPH) changed to hyperaemia, 3 with IPH moved to low flow, 1 with IPH subsequently developed middle cerebral artery vasospasm, and 2 with low flow transitioned to vasospasm. TCD phenotype did not change in the remaining children. Normalization of flow velocities and morphology occurred at significantly different time points depending on the underlying phenotype (Fig. 2). By hospital day 2, 97% of children with MO, 63% with hyperaemia, and 67% with IPH had normalized their TCD findings, whereas only 42% of those with low flow and 22% of those with vasospasm had (p = 0.02). By day 4, most surviving children in each phenotype had normalized (hyperaemia 85%, IPH 93%, Low flow 85%, MO 100%, Vasospasm 95%. p = 0.67). Three children with low flow and one with vasospasm had not normalized TCD by hospital day 8. Figure 3 displays representative images of children classified into each of the phenotypes.

Differences in demographics, physical examination, laboratory results, and imaging findings in children with different TCD phenotypes are in Table 4. Plasma *Plasmodium falciparum* histidine rich protein (*Pf*HRP2) was lowest in children meeting criteria for the microvascular obstruction phenotype (p = 0.006). The pulse pressure, or the difference between the systolic and diastolic blood pressure, was significantly higher in children with hyperaemia (median pulse pressure 43 [39, 55] mmHg) than other phenotypes (p = 0.003). The stroke volume index was on the low end of normal or reduced compared to published values for age (40–55 ml/m2) in all groups. While not statistically significant, there was a trend to children with low flow also having the lowest SVI (SVI 32 [28.5,41], p = 0.08) [36]. For most phenotypes, the cardiac index (CI) was generally above the published normal range (CI 3.5–5 l/min/m^2^) and systemic vascular resistive index was normal (SVRI 1000–1600 d.s.cm-^5^m^2^). Children with low flow had significantly lower CI and higher SVRI than other groups (median CI 4.4 [3.7,5] l/min/m^2^ (p =  < 0.001), median SVRI 1552 [1197, 1961] d.s.cm^−5^m^2^ (p = 0.003)). No other statistically significant differences of known contributors to TCD changes (outlined in Table 1) were identified between TCD phenotypic groups.

**Table 4:** Demographics, laboratory investigations, imaging, and outcomes by Transcranial Doppler Ultrasound phenotype

Variable	Hyperemia (n = 57)	IPH (n = 35)	Low flow (n = 50)	MO (n = 14)	Vasospasm (n = 11)	Normal (n = 7)	*p*
Age (months), mean (SD)	55 (31)	58 (37)	59 (37)	56 (25)	67 (32)	72 (29)	0.71
Temperature (°C), median [IQR]	38.5 [37.7, 39.1]	38.5 [37.8, 39.4]	38.0 [37.1, 39.2]	38.5 [37.6, 39.3]	37.8 [37.6, 38.5]	38.5 [38.0, 39.0]	0.25
Heart rate (beats/min), median [IQR]	141 [127, 152]	143 [128, 158]	142 [123, 161]	140 [124, 158]	140 [136, 146]	133 [130, 136]	0.97
RR (breaths/min), median [IQR]	32.0 [26.0, 39.0]	36.0 [28.0, 43.0]	36.0 [32.0, 42.8]	34.5 [32.2, 39.5]	36.0 [32.5, 44.5]	40.0 [39.5, 45.0]	0.07
Oxygen saturation (%), median [IQR]	97.0 [95.0, 98.0]	97.0 [96.0, 99.0]	97.0 [95.0, 98.0]	98.0 [96.2, 98.0]	98.0 [97.0, 99.0]	96.0 [96.0, 97.5]	0.21
MBP (mmHg), median [IQR]	78.0 [71.0, 84.0]	78.0 [72.5, 86.5]	83.5 [73.2, 90.0]	73.0 [65.8, 80.5]	75.0 [68.5, 86.5]	74.0 [71.0, 79.0]	0.21
Pulse pressure (mmHg), median [IQR]	43.0 [39.0, 55.0]	38.0 [35.0, 44.0]	37.5 [33.0, 44.8]	35.5 [34.0, 43.2]	38.0 [35.0, 42.0]	33.0 [33.0, 35.5]	0.003
Stroke volume index (ml/m^2^), median [IQR]	39.0 [34.2, 44.8]	39.0 [33.5, 43.5]	32.0 [28.5, 41.0]	43.0 [38.2, 50.8]	42.0 [38.2, 46.2]	38.0 [35.0, 42.5]	0.08
Cardiac index (l/min/m^2^), median [IQR]	5.30 [4.90, 5.88]	5.40 [4.55, 5.90]	4.40 [3.70, 5.00]	6.00 [5.18, 6.60]	5.55 [5.20, 6.60]	4.20 [4.00, 5.05]	< 0.001
SVRI (d.s.cm^−5^m^2^), median [IQR]	1270 [1070, 1392]	1306 [1198, 1580]	1552 [1197, 1961]	962 [919, 991]	1187 [903, 1334]	1378 [1294, 1660]	0.003
Packed Cell Volume (%),median [IQR]	26.0 [20.0, 29.0]	26.0 [21.5, 30.0]	27.5 [22.2, 32.8]	27.5 [24.2, 30.8]	23.0 [19.5, 25.0]	27.0 [25.0, 28.0]	0.08
Glucose (mmol/L), median [IQR]	5.30 [4.40, 6.20]	5.90 [4.60, 6.60]	5.65 [4.40, 6.90]	5.30 [4.38, 8.85]	5.80 [5.40, 6.60]	5.70 [5.15, 6.50]	0.74
Lactate (mmol/L), median [IQR]	4.30 [2.60, 6.80]	3.00 [2.10, 5.30]	4.05 [2.02, 8.60]	4.15 [2.10, 6.95]	5.00 [2.75, 8.35]	4.00 [3.00, 4.70]	0.87
Parasites/microliter blood, median [IQR]	455,000 [27,000, 975,000]	346,000 [108,000, 1,499,000]	464,000 [180,000 1,280,000]	126,000 [67,000, 459,000]	116,000 [62,000, 237,000]	246,000 [174,000, 815,000]	0.52
*Pf*HRP2 (ng/mL), median [IQR]	396 [111,1021]	913 [46, 1270]	333 [122, 874]	123 [13, 282]	330 [44,687]	807 [545, 996]	.006
pH, median [IQR]	7.42 [7.36, 7.46]	7.42 [7.36, 7.45]	7.41 [7.38, 7.46]	7.44 [7.36, 7.48]	7.38 [7.35, 7.50]	7.41 [7.38, 7.44]	0.99
CO2 (mmHg), median [IQR]	29.0 [23.0, 36.0]	28.5 [24.8, 34.0]	27.5 [24.0, 31.0]	27.0 [24.5, 30.5]	22.5 [18.0, 26.5]	26.0 [25.5, 34.5]	0.53
Base excess, median [IQR]	2.10 [-3.00, 6.50]	0.80 [-6.50, 6.00]	0.10 [-6.00, 5.00]	3.30 [2.70, 7.82]	3.00 [0.50, 20.2]	5.00 [1.50, 6.00]	0.22
Sodium (mEq/L), median [IQR]	139 [137, 143]	139 [136, 144]	140 [135, 143]	136 [134, 139]	138 [134, 140]	141 [137, 146]	0.29
Bicarbonate (mmol/L), median [IQR]	17.0 [13.0, 20.2]	17.0 [15.0, 19.0]	18.0 [13.5, 20.5]	17.8 [12.2, 20.0]	18.0 [13.5, 21.5]	18.5 [17.8, 20.0]	0.93
Retinopathy positive, n (%)	37 (65)	21 (60)	36 (72)	10 (71.4)	6 (54.5)	5 (71.4)	0.80
Blantyre coma score, n (%)							0.05
0	7 (13)	5 (14)	8 (16)	4 (29)	2 (18)	0 (0)	
1	15 (26)	14 (40)	26 (52)	3 (21)	3 (27)	3 (43)	
2	35 (61)	16 (46)	16 (32)	7 (50)	6 (55)	4 (57)	
Seizures on EEG, n (%)	6 (11)	1 (3)	4 (8)	3 (21)	2 (18)	0 (0)	0.38
Papilloedema present, n (%)	3 (5)	1 (3)	3 (6)	2 (14)	1 (9)	0 (0)	0.37
Opening pressure (cm H20), median [IQR]	15.5 [12.0, 21.5]	16.0 [13.5, 21.5]	17.0 [12.0, 22.0]	18.0 [14.0, 19.0]	21.5 [12.8, 26.0]	19.0 [12.0, 23.5]	0.83
ONSD (mm), median [IQR]	0.48 [0.44, 0.53]	0.48 [0.44, 0.50]	0.48 [0.45, 0.50]	0.49 [0.45, 0.53]	0.45 [0.42, 0.48]	0.46 [0.44, 0.47]	0.59
Time to coma resolution (hrs), median [IQR]	26.0 [20.0, 61.5]	44.0 [24.0, 79.5]	52.0 [25.5, 90.5]	40.0 [31.0, 51.0]	47.0 [19.5, 114]	36.0 [27.0, 36.0]	0.23
Brain volume score, n (%)*	n = 34	n = 17	n = 24	n = 10	n = 7	n = 2	0.95
3	3 (9)	1 (6)	1 (4)	1 (10)	1 (14)	0 (0)	
4	3 (9)	1 (6)	3 (13)	1 (10)	0 (0)	0 (0)	
5	13 (38)	6 (35)	8 (33)	3 (30)	3 (43)	0 (0)	
6	9 (26)	4 (24)	5 (21)	2 (20)	2 (29)	2 (100)	
7	5 (15)	5 (29)	5 (21)	2 (20)	1 (14)	0 (0)	
8	1 (3)	0 (0)	2 (8)	1 (10)	0 (0)	0 (0)	
Autoregulation (THRR), median [IQR]	1.05 [1.01, 1.12]	1.11 [1.07, 1.19]	1.10 [1.03, 1.20]	1.09 [1.07, 1.13]	1.04 [1.01, 1.06]	1.20 [1.14;1.27]	0.13
Outcome, n (%)							< .001
Good							
PCPC 1 -2 (normal, mild disability)	44 (77)	31 (89)	21 (42)	9 (64)	6 (55)	7 (100)	
Poor							
PCPC 3–5 (moderate to severe disability)	8 (14)	3(9)	14 (28)	3 (21)	5 (45)	0 (0)	
PCPC 6 (died)	5 (9)	1 (2)	15 (30)	2 (15)	0 (0)	0 (0)	

*n* number, *SD* standard deviations, *hrs* hours, *RR* respiratory rate, *SBP* systolic blood pressure, *mmHg* millimeters mercury, *DBP* diastolic blood pressure; *MBP* mean blood pressure, *IQR* interquartile range, *SVRI* systemic vascular resistive index, *PfHRP2* Plasmodium falciparum histidine rich protein 2, *CO*_2_ carbon dioxide, *NIRS* near-infrared spectroscopy, *SO*_2_ cerebral oxygen saturation, *EEG* electroencephalogram, *OP* opening pressure, *ONSD* optic nerve sheath diameter, *THRR* transient hyperemic response ratio, *MRI* magnetic resonance imaging, *TCD* transcranial doppler ultrasound, *CSF* cerebrospinal fluid

MRI data are available for 94 participants

### Outcomes

Overall, 118 children (68%) had a good neurologicalal outcome at the time of hospital discharge. Twenty-three (13%) died, and 33 (19%) had moderate to severe deficits (Table 3). Outcomes were best for participants with TCD-defined hyperaemia and IPH (PCPC 1–2 in 77 and 89% respectively). Participants with TCD-defined low flow had the highest day 1 mortality and the least likelihood of a good outcome (PCPC 1–2 in 42%) (p < 0.001) (Tables 4, 5). Cerebral autoregulation was significantly better in children with good outcome (THRR 1.12 [1.04,1.2]) compared to a poor outcome (THRR 1.05 [0.98,1.02], p = 0.046).

**Table 5:** Number of deaths by day by Transcranial Doppler Ultrasound phenotype

TCD Phenotype	Post admission day	Number of deaths	% of overall deaths on that day
Hyperaemia	1	1	8%
	2	2	50%
	3	1	25%
	4	1	100%
Isolated posterior high flow	1	1	8%
Low flow	1	10	76%
	2	2	50%
	3	2	50%
	4	0	0%
	5	1	100%
Microvascular obstruction	1	1	8%
	2	0	0%
	3	1	25%

## Discussion

Previous work in the Democratic Republic of the Congo (DRC) described five different Transcranial Doppler Ultrasound phenotypes in a cohort of children with CM [30]. The current study, in a unique group of children with CM in Malawi, identified the same five phenotypes in similar proportions to what was previously reported: hyperaemia (28% in DRC, 33% in Malawi), low flow (28% in DRC, 29% in Malawi), microvascular obstruction (23% in DRC, 8% in Malawi), vasospasm (14% in DRC, 6% in Malawi), and isolated posterior high flow (7% in DRC, 20% in Malawi). In both studies, hyperaemia was associated with a higher likelihood of favourable outcome whereas low flow was associated with increased mortality. Impaired autoregulation was also identified in both studies as being significantly associated with worse outcomes. It is unusual to identify multiple distinct changes to TCD flow velocities and morphology in a clinical diagnosis with a single underlying pathologic mechanism. Thus, given the number of phenotypes again identified in this population of children with CM, the hypothesis that multiple different mechanisms contribute to neurological injury and neuroimaging findings in CM must be considered.

Cerebral blood flow (CBF), and thus TCD flow velocities and waveforms, are dependent on cerebral perfusion pressure (CPP) and inversely proportional to the cerebrovascular resistance (CVR) [15]. CPP is determined by the pressure gradient between the brain’s supplying arteries (mean arterial pressure, MAP) and the central venous pressure, which is approximately equivalent to the intracranial pressure (ICP) so that: CBF = CPP/CVR = (MAP − ICP)/CVR. Thus, increases in MAP may result in increased CBF, particularly if the blood pressure is elevated above the autoregulatory threshold OR if less elevated but autoregulation is impaired. Low MAP has the opposite effect on CBF, which again, may be particularly significant when blood pressures fall below the lower limit of autoregulatory capacity or if autoregulation is not intact. When hyperaemia is identified on TCD, significant hypertension or elevated blood pressures with impaired autoregulation should be considered as potential mechanisms of that phenotype. Hypotension or relatively low blood pressures with impaired autoregulation may result in a low flow phenotype on TCD.

Increases in ICP will also alter CBF. With mild to moderate elevations in ICP, normal systolic flow is generally maintained but a preferential reduction in diastolic blood flow occurs as small cerebral vessels are compressed. This results in a high pulsatility index identified on TCD (with the combination of these alterations equating to the “microvascular obstruction” phenotype described in this study). Significant intracranial hypertension can result in low flow of all measured velocities accompanied by characteristic alterations to the TCD waveform (systolic spikes and absent or reversal of diastolic flow).

The CVR is determined by the smooth muscle tone of the cerebral vessels. This tone is controlled by a multiplicity of components that cross talk to maintain brain homeostasis over a range of physiologic conditions and in response to changing cerebral metabolic demand [16-2116-21]. For example, blood viscosity is inversely related to CBF; reduction of shear force applied to the cerebrovascular endothelium as viscosity falls reduces CVR and CBF increases. Thus, anaemia can result in increased CBF and be identified as hyperaemia on TCD. Hypoxia increases endothelial production of vasodilating substances, reduces CVR, and increases CBF, again resulting in the hyperaemia phenotype on TCD. Hypercapnia and hypocapnia, likely through modulating nitric oxide, decrease and increase CVR respectively. As such, hypercapnia is frequently identified as hyperaemia on TCD and hypocapnia as reduced diastolic flow and increased PI (“microvascular obstruction/alteration” in this study). Additionally, circulating, parenchymal, and endothelially derived vasodilatory and vasoconstricting compounds alter vascular tone/CVR and increase or decrease CBF to meet metabolic demand locally. Thus, fever or seizures that increase demand will increase production of vasodilatory compounds and result in hyperaemia on TCD.

These classic physiologic or pathologic factors that contribute to specific TCD flow velocity or waveform alterations in most situations (Table 1) were not clearly causative of the identified phenotypes in children with CM. Patients categorized as having hyperaemia and isolated posterior circulation high flow were not more hypertensive, anaemic, hypercapnic, febrile, or more likely to be having seizures than those classified into a different TCD phenotype (Table 4). Those with low flow were not more likely to have indirect evidence of significant increased intracranial pressure (ICP) (opening pressure on lumbar puncture, optic nerve sheath diameter, or brain volume score) than other phenotypes. Hypocapnia/alkalosis and signs of early increased intracranial pressure were no more likely in children with microvascular obstruction than other phenotypes.

Differences in some cardiovascular parameters from normal as well as between TCD phenotypic groups were observed. Across the cohort, cardiac index (CI) was within or above the published normal value for age whereas stroke volume index (SVI) was at the low end of normal or reduced. CI is calculated as CI = Heart rate x SVI and SVI as SVI = End diastolic volume – End systolic volume. Low SVI in CM patients likely represents decreased preload (and hence reduced end diastolic volume) secondary to some component of decreased circulating blood volume and/or dehydration [37]. A compensatory increase in heart rate maintains or increases CI to meet high systemic metabolic demands, thus explaining the normal to elevated CI identified in the cohort. SVI and CI were lowest in children with the low flow phenotype, potentially due to greater reductions in preload than in other groups. Additionally, systemic vascular resistance was highest in children categorized as low flow, which by increasing end systolic volume, could have also contributed to the lower CI in this group. However, CI was still within the normal range for age in children identified as having low flow on TCD, decreasing the likelihood that poor cardiac output completely contributed to the low flow velocities observed in the cerebrovasculature.

Therefore, alternative potential mechanisms leading to TCD phenotypes in paediatric CM must be considered. Examination of the brain tissue of children who have died of CM reveals sequestration, a multifocal microvascular obstruction by adherent, parasitized red blood cells [38]. Sequestration results in endothelial cell activation, increased cytokine production, neurovascular inflammation, and blood–brain barrier disruption [39]. Neuroinflammation is known to affect multiple metabolic pathways in the central nervous system [40–45]. Overactivation or dysregulation of these metabolic pathways in the central nervous system may result in the accumulation or depletion of local circulating, parenchymal, or endothelially derived vasoactive compounds. These factors may contribute to observed TCD phenotypes through the alteration of neurovascular tone [46–57]. Future work should examine the relationships between potential putative compounds and TCD phenotypes.

## Conclusions

TCD identified multiple different flow velocity and waveform alterations across a cohort of children with CM, each associated with unique outcomes. Common pathohysiological mechanisms associated with TCD phenotypes in non-malarial illness were not clearly identified as causative in children with CM. Alternative mechanistic contributors, including mechanical factors of the cerebral circulation and/or biologically active regulators of vascular tone, should be explored. If identified, TCD could then be used as a point of care tool to optimize individual cerebral physiology through targeted adjunctive interventions.

## Data Availability

Not applicable.

## References

[1] WHO. World Malaria Report. Geneva: World Health Organization, 2020. Available at: http://www.who.int /malaria/publications/world_ malaria_report/en. Accessed April 15, 2021.

[2] Seydel KB, Kampondeni SD, Valim C, Potchen MJ, Milner DA, Muwalo FW (2015). Brain swelling and death in children with cerebral malaria. N Eng J Med.

[3] Idro R, Jenkins NE, Newton C (2005). Pathogenesis, clinical features and neurological outcome of cerebral malaria. Lancet Neurol.

[4] Idro R, Marsh K, John CC, Newton CRJ (2010). Cerebral malaria; mechanisms of brain injury and strategies for improved neuro-cognitive outcome. Pediatr Res.

[5] Langfitt JT, McDermott MP, Brim R, Mboma S, Potchen MJ, Kampondeni SD (2019). Neurodevelopmental impairments 1year after cerebral malaria. Pediatrics.

[6] Birbeck GL, Molyneux ME, Kaplan PW, Seydel KB, Chimalizeni YF, Kawaza K (2010). Blantyre Malaria Project Epilepsy Study (BMPES) of neurological outcomes in retinopathy-positive pediatric cerebral malaria survivors: a prospective cohort study. Lancet Neurol.

[7] Potchen MJ, Kampondeni SD, Seydel KB, Haake CK, Sinyangwe SS, Mwenechanya M, et al. 1.5 Tesla magnetic resonance imaging to investigate potential etiologies of brain swelling in pediatric cerebral malaria. Am J Trop Med Hyg. 2018;98:497–504. 10.4269/ajtmh.17-0309 PMC5929182 29313473

[8] Potchen MJ, Birbeck GL, DeMarco JK, Kampondeni SD, Beare N, Molyneux ME (2010). Neuroimaging findings in children with retinopathy confirmed cerebral malaria. Eur J Radiol.

[9] Aaslid R, Markwalder TM, Nornes H (1982). Noninvasive transcranial Doppler ultrasound recording of flow velocity in basal cerebral arteries. J Neurosurg.

[10] Bode H, Wais U (1988). Age dependence of flow velocities in basal cerebral arteries. Arch Dis Child.

[11] Lindegaard KF, Nornes H, Bakke SJ, Sorteberg W, Nakstad P (1989). Cerebral vasospasm diagnosis by means of angiography and blood velocity measurements. Acta Neurochir.

[12] Aaslid R, Lindegaard KF, Sorteberg W, Nornes H (1989). Cerebral autoregulation dynamics in humans. Stroke.

[13] LaRovere KL, Tasker RC, Wainwright M, Reuter-Rice K, Appavu B, Miles D (2020). Transcranial Doppler Ultrasound during critical illness in children: survey of practices in pediatric neurocritical care centers. PCCM.

[14] Verlhac S (2011). Transcranial Doppler in children. Pediatr Radiol.

[15] Silverman A, Petersen NH. Physiology, cerebral autoregulation. In: StatPearls [Internet]. Treasure Island (FL): StatPearls Publishing; 2021. Available from: https://www.ncbi.nlm.nih.gov/books/NBK553183/31985976

[16] Roloff EV, Tomiak-Baquero AM, Kasparov S, Paton JF (2016). Parasympathetic innervation of vertebrobasilar arteries: is this a potential clinical target?. J Physiol.

[17] Iida N, Mitamura Y (1989). Effects of venous pressure elevation on myogenic vasoconstrictive responses to static and dynamic arterial pressures. Jpn J Physiol.

[18] Boedtkjer E (2018). Acid-base regulation and sensing: accelerators and brakes in metabolic regulation of cerebrovascular tone. J Cereb Blood Flow Metab.

[19] Pearce WJ (1995). Mechanisms of hypoxic cerebral vasodilatation. Pharmacol Ther.

[20] Morita Y, Hardebo JE, Bouskela E (1994). Influence of cerebrovascular parasympathetic nerves on resting cerebral blood flow, spontaneous vasomotion, autoregulation, hypercapnic vasodilation and sympathetic vasoconstriction. J Auton Nerv Syst.

[21] Morita-Tsuzuki Y, Hardebo JE, Bouskela E (1993). Interaction between cerebrovascular sympathetic, parasympathetic and sensory nerves in blood flow regulation. J Vasc Res.

[22] Spencer MP (1997). Transcranial Doppler monitoring and causes of stroke from carotid endarterectomy. Stroke.

[23] Zidan DH, Helmy TA, Taha A (2017). Role of transcranial Doppler and FOUR score in assessment of sepsis-associated encephalopathy. Res Opin Anesth Intensive Care.

[24] Thorpe SG, Thibeault CM, Canac N, Jelaliddini K, Dorn A, Wilk SJ (2020). Toward automated classification of pathological transcranial Doppler waveform morphology via spectral clustering. PLoS ONE.

[25] Burgin WS, Malkoff M, Felberg RA, Demchuk AM, Christou I, Grotta JC (2000). Transcranial doppler ultrasound criteria for recanalization after thrombolysis for middle cerebral artery stroke. Stroke.

[26] Demchuk AM, Burgin WS, Christou I, Felberg RA, Barber PA, Hill MD (2001). Thrombolysis in Brain Ischemia (TIBI) transcranial Doppler flow grades predict clinical severity, early recovery, and mortality in patients treated with tissue plasminogen activator. Stroke.

[27] Thorpe SG, Thibeault CM, Wilk SJ, O'Brien M, Canac N, Ranjbaran M (2019). Velocity curvature index: a novel diagnostic biomarker for large vessel occlusion. Transl Stroke Res.

[28] Thorpe SG, Thibeault CM, Canac N, Wilk SJ, Devlin T, Hamilton R (2018). Decision decision criteria for large vessel occlusion using Transcranial Doppler Waveform morphology. Front Neurol.

[29] Alexandrov AV, Sloan MA, Tegeler CH, Newell DN, Lumsden A, Garami Z, et al. American Society of Neuroimaging Practice Guidelines Committee. Practice standards for transcranial Doppler (TCD) ultrasound. Part II. Clinical indications and expected outcomes. J Neuroimaging. 2012;22:215–24. 10.1111/j.1552-6569.2010.00523.x 20977531

[30] O’Brien NF, Tshimanga MT, Moore-Clingenpeel MM, Bodi Mabiala J, Pongo JM, Musungufu DA (2018). Transcranial Doppler Ultrasonography provides insights into neurovascular changes in children with cerebral malaria. J Pediatrics.

[31] Beare NA, Taylor TE, Harding SP, Lewallen S, Molyneux ME (2006). Malarial retinopathy: a newly established diagnostic sign in severe malaria. Am J Trop Med Hyg.

[32] Taylor TE (2009). Caring for children with cerebral malaria: insights gleaned from 20 years on a research ward in Malawi. Trans R Soc Trop Med Hyg.

[33] Barrow GI, Feltham RKA (1993). Cowan and Steel’s manual for the identification of medical bacteria.

[34] Fiser DH (1992). Assessing the outcome of pediatric intensive care. J Pediatr.

[35] Fiser DH, Tilford JM, Roberson PK (2000). Relationship of illness severity and length of stay to functional outcomes in the paediatric intensive care unit: a multi-institutional study. Crit Care Med.

[36] Cattermole GN, Leung PY, Mak PS, Chan SS, Graham CA, Rainer TH (2010). The normal ranges of cardiovascular parameters in children measured using the Ultrasonic Cardiac Output Monitor. Crit Care Med.

[37] English MC, Waruiru C, Lightowler C, Murphy SA, Kirigha G, Marsh K (1996). Hyponatraemia and dehydration in severe malaria. Arch Dis Child.

[38] Taylor TE, Fu WJ, Carr RA, Whitten RO, Mueller JS, Fosiko NG (2004). Differentiating the pathologies of cerebral malaria by postmortem parasite counts. Nat Med.

[39] Faille D, El-Assaad F, Alessi MC, Fusai T, Combes V, Grau GE (2009). Platelet-endothelial cell interactions in cerebral malaria: the end of a cordial understanding. Thromb Haemost.

[40] Yan J, Kuzhiumparambil U, Bandodkar A, Bandodkar S, Dale RC, Fu S (2021). Cerebrospinal fluid metabolites in tryptophan-kynurenine and nitric oxide pathways: biomarkers for acute neuroinflammation. Dev Med Child Neurol.

[41] Yan J, Kuzhiumparambil U, Bandodkar S, Dale RC, Fu S (2021). Cerebrospinal fluid metabolomics: detection of neuroinflammation in human central nervous system disease. Clin Transl Immunology.

[42] Davis I, Liu A (2015). What is the tryptophan kynurenine pathway and why is it important to neurotherapeutics?. Expert Rev Neurother.

[43] Bipath P, Levay PF, Viljoen M (2015). The kynurenine pathway activities in a sub-Saharan HIV/AIDS population. BMC Infect Dis.

[44] Troché G, Henry-Lagarrigue M, Soppelsa F, Legriel S, Yehia A, Bruneel F (2020). Tryptophan pathway catabolites (serotonin, 5-hydroxyindolacetic acid, kynurenine) and enzymes (monoamine oxidase and indole amine 2,3 dioxygenase) in patients with septic shock: A prospective observational study versus healthy controls. Medicine (Baltimore).

[45] Lugo-Huitrón R, Ugalde Muñiz P, Pineda B, Pedraza-Chaverrí J, Ríos C, Pérez-de la Cruz V. Quinolinic acid: an endogenous neurotoxin with multiple targets. Oxid Med Cell Longev. 2013;2013:104024. 10.1155/2013/104024 PMC3780648 24089628

[46] Tao KM, Li XQ, Yang LQ, Yu WF, Lu ZJ, Sun YM, et al. Glutamine supplementation for critically ill adults. Cochrane Database Syst Rev. 2014;9:CD010050. 10.1002/14651858.CD010050.pub2 PMC6517119 25199493

[47] Morris CR, Hamilton-Reeves J, Martindale RG, Sarav M, Ochoa Gautier JB (2017). Acquired amino acid deficiencies: a focus on arginine and glutamine. Nutr Clin Pract.

[48] Kelly D, Wischmeyer PE (2003). Role of L-glutamine in critical illness: new insights. Curr Opin Clin Nutr Metab Care.

[49] Dobbs KR, Embury P, Vulule J, Odada PS, Rosa BA, Mitreva M (2017). Monocyte dysregulation and systemic inflammation during pediatric falciparum malaria. JCI Insight.

[50] Anstey NM, Weinberg JB, Hassanali MY, Mwaikambo ED, Manyenga D, Misukonis MA (1996). Nitric oxide in Tanzanian children with malaria: inverse relationship between malaria severity and nitric oxide production/nitric oxide synthase type 2 expression. J Exp Med.

[51] Oyegue-Liabagui SL, Bouopda-Tuedom AG, Kouna LC, Maghendji-Nzondo S, Nzoughe H, Tchitoula-Makaya N (2017). Pro- and anti-inflammatory cytokines in children with malaria in Franceville. Gabon Am J Clin Exp Immunol.

[52] Hobbs MR, Udhayakumar V, Levesque MC, Booth J, Roberts JM, Tkachuk AN (2002). A new NOS2 promoter polymorphism associated with increased nitric oxide production and protection from severe malaria in Tanzanian and Kenyan children. Lancet.

[53] Lopansri BK, Anstey NM, Weinberg JB, Stoddard GJ, Hobbs MR, Levesque MC (2003). Low plasma arginine concentrations in children with cerebral malaria and decreased nitric oxide production. Lancet.

[54] Medana IM, Day NPJ, Houta SS, Stocker R, Smythe G, Bwanaisa L (2003). Metabolites of the kynurenine pathway of tryptophan metabolism in the cerebrospinal fluid of Malawian children with malaria. JID.

[55] Crowley RW, Medel R, Kassell NF, Dumont AS (2008). New insights into the causes and therapy of cerebral vasospasm following subarachnoid hemorrhage. Drug Discov Today.

[56] Rubach MP, Zhang H, Florence SM, Mukemba JP, Kalingonji AR, Anstey NM (2019). Kinetic and cross-sectional studies on the genesis of hypoargininemia in severe paediatric *Plasmodium falciparum* malaria. Infect Immun.

[57] Weinberg JB, Yeo TW, Mukemba JP, Florence SM, Volkheimer AD, Wang H (2014). Dimethylarginines: endogenous inhibitors of nitric oxide synthesis in children with falciparum malaria. J Infect Dis.

